# P40 and P90 from Mpn142 are Targets of Multiple Processing Events on the Surface of *Mycoplasma pneumoniae*

**DOI:** 10.3390/proteomes3040512

**Published:** 2015-12-16

**Authors:** Michael Widjaja, Iain J. Berry, Elsa J. Pont, Matthew P. Padula, Steven P. Djordjevic

**Affiliations:** 1 ithree institute, University of Technology Sydney, P.O. Box 123 Broadway, Ultimo, NSW 2007, Australia; E-Mails: michael.widjaja@student.uts.edu.au (M.W.); iain.j.berry@student.uts.edu.au (I.J.B.); elsa.pont@etudiant.univ-lille1.fr (E.J.P.); 2 Proteomics Core Facility, University of Technology Sydney, Cnr Harris and Thomas St, Ultimo, NSW 2007, Australia; E-Mail: matthew.padula@uts.edu.au

**Keywords:** ectodomain shedding, protein processing, multifunctional proteins, *Mycoplasma pneumoniae* M129 strain, P40 and P90, proteins B and C, Mpn142, protein disorder, endoproteolysis

## Abstract

*Mycoplasma pneumoniae* is a significant cause of community acquired pneumonia globally. Despite having a genome less than 1 Mb in size, *M. pneumoniae* presents a structurally sophisticated attachment organelle that (i) provides cell polarity, (ii) directs adherence to receptors presented on respiratory epithelium, and (iii) plays a major role in cell motility. The major adhesins, P1 (*Mpn141*) and P30 (*Mpn453*), are localised to the tip of the attachment organelle by the surface accessible cleavage fragments P90 and P40 derived from Mpn142. Two events play a defining role in the formation of P90 and P40; removal of a leader peptide at position 26 (^23^SLA↓NTY^28^) during secretion to the cell surface and cleavage at amino acid 455 (^452^GPL↓RAG^457^) generating P40 and P90. Liquid Chromatography Tandem Mass Spectrometry (LC-MS/MS) analysis of tryptic peptides generated by digesting size-fractionated cell lysates of *M. pneumoniae* identified 15 cleavage fragments of Mpn142 ranging in mass from 9–84 kDa. Further evidence for the existence of cleavage fragments of Mpn142 was generated by mapping tryptic peptides to proteins recovered from size fractionated eluents from affinity columns loaded with heparin, fibronectin, fetuin, actin, plasminogen and A549 surface proteins as bait. To define the sites of cleavage in Mpn142, neo-N-termini in cell lysates of *M. pneumoniae* were dimethyl-labelled and characterised by LC-MS/MS. Our data suggests that Mpn142 is cleaved to generate adhesins that are auxiliary to P1 and P30.

## 1. Introduction

*Mycoplasma pneumoniae* (*M. pneumoniae*) is a respiratory pathogen estimated to cause 100,000 hospitalisations annually in the USA [1] and up to 40% of cases of community-acquired pneumonia globally [2]. *M. pneumoniae* infects patients of all ages and up to 25% of affected individuals develop extrapulmonary complications at neurological, musculoskeletal, haematological and cardiovascular sites. While sporadic infections are common, outbreaks of *M. pneumoniae-*induced disease occur in schools, child-care facilities, inpatient institutions and military barracks [3]. Azithromycin and other macrolides are used for the treatment of infections caused by *M. pneumoniae* [4], but an increase in the frequency of reports of macrolide resistance, particularly in Europe, Asia and North America, is of major concern [5,6,7,8,9,10,11,12,13,14,15,16,17,18,19]. Efficacious vaccines for the prevention of infections caused by *M. pneumoniae* are yet to be developed and are complicated by the presence of antigens that are capable of evoking an autoimmune response [20]. While the infiltration of neutrophils and lymphocytes is a characteristic immunological hallmark of infections caused by *M. pneumoniae*, the severity of the response varies widely. In severe cases, the immune response is known to generate immunopathological sequelae, complicating vaccine design [2,20].

*M. pneumoniae* has a small genome encoding about 700 ORFs and lacks genes needed for a TCA cycle, and cell wall, amino acid and nucleotide biosynthesis [21,22]. Despite having a reduced genome capacity, *M. pneumoniae* is remarkable in that it forms a Triton X-100 insoluble cytoskeleton and complex attachment organelle that is critical for adherence to host epithelium and cellular motility [23]. The attachment organelle comprises the adhesins P1 and P30, High Molecular Weight (HMW) proteins 1, 2 and 3, P40/P90 from *mpn142* (ORF6), P65, P41 and P24 [24,25,26,27,28,29]. Adhesins P1, P30 and Mpn142 products (P40/P90) are strictly localised to the extracellular side of the attachment organelle and have N-terminal transmembrane domains that form part of a signal sequence [30,31,32,33,34]. P65 and HMW1 are unusual because they reside intracellularly as part of the cytoskeletal core and on the extracellular side of the attachment organelle, suggesting the existence of different proteoforms [25]. It is not known how HMW1 traffics to the cell surface, because it lacks evidence of transmembrane spanning domains and a secretion signal. HMW 1, 2 and 3, P41, P24 and P65 are all integral components of the intracellular cytoskeletal core [35].

Layh-Schmitt *et al.* [24] described the use of para-formalydehyde to crosslink proteins in close association with one another and identified protein complexes containing the P1 adhesin by affinity chromatography using P1 antibodies. The identities of the proteins in the complex were determined by a combination of immunoblot analysis and MALDI-TOF MS. P1 complexes contained P40 and P90, P30, P65, DnaK, pyruvate dehydrogenase subunit α, HMW1 and HMW3 proteins [24]. Notably, the P1 adhesin was found to be associated with P30, P40 and P90 when *M. pneumoniae* cells were treated with the membrane impermeable cross-linking reagent DTSSP (3,3ʹ-dithiobis(sulfosuccinimidyl proprionate)) suggesting that some of the interactions with P1 are not accessible on the extracellular side of the membrane [36]. Notably, a 480 kDa protein complex was isolated by solubilising *M. pneumoniae* proteins after cross-linking with bis(sulfosuccinimidyl) suberate (BS^3^) using a non-ionic detergent and Blue Native PAGE. The complex comprises P90 and P1 in a 1:2 molar ratio and forms an appendage that allows *M. pneumoniae* to glide across surfaces [37].

While expression of P1 is essential for adherence, the presence of accessory adherence proteins is critical for the formation of a functional attachment organelle [25]. Insertion of P1 into the membrane and its trafficking to the attachment organelle is largely dependent on P90 and P40 [38,39,40]. Mutants defective in the expression of P90 and P40 allow P1 to completely partition to the Triton X-100 soluble phase. In wild type cells, P1 typically partially associates with the Triton X-100 insoluble shell [39]. Mutants unable to produce *mpn142* are defective in cellular adherence because P1 cannot traffic to the tip structure resulting in random P1 distribution around the cell body [38,40]. The P1 and P30 adhesins concentrate at the tip of the attachment organelle and represent the dominant proteins responsible for adherence [38,41,42,43,44,45,46]. Mutants that express P1 and P30 but that lack P40/P90, or the HMW proteins 1, 2 and 3, are avirulent. As such P1 and P30 are considered to be essential but not sufficient for attachment of *M. pneumoniae* to host cells [47,48,49].

*mpn140*-*mpn141*-*mpn142* comprise a polycistronic transcriptional unit presumably to ensure equimolar amounts of each of the proteins [50,51]. Mpn140 encodes for a putative phosphoesterase of 28 kDa that has been found to be expressed but remains functionally uncharacterised [50,52,53,54]. *mpn141* encodes the P1 adhesin, a 170 kDa protein comprised of 1627 amino acids [31] while Mpn142 encodes a 130 kDa adherence accessory protein comprising 1218 amino acids [52]. The function of Mpn142 is complicated by the fact that it undergoes processing shortly after translation at two sites generating an N-terminal 40 kDa protein (P40/protein C) and a C-terminal 90 kDa protein (P90/protein B) [33,52]. Notably, the predicted 130 kDa precursor is not detectable indicating that the cleavage events are efficient [33,52]. P90 and P40 were able to be linked to the P1 protein via a non-permeating, cross-linking reagent indicating that all three proteins co-localise on the extracellular side of the membrane on the tip of the attachment organelle within a distance of approximately 12 Å (1.2 nm) to one other [36]. *M. pneumoniae* mutant strain M29-B176 lacks P40 and P90 proteins and is avirulent [49]; other *M. pneumoniae* mutants that are unable to express P40 and P90 are also avirulent [40,55,56].

Leader sequences in the N-terminus of P1 and Mpn142 are removed by cleavage at positions 60 and 26, respectively [30,34,57]. Cleavage at position 26 in Mpn142 was confirmed by identifying the semi-tryptic peptide ^26^NTYLLQDHNTLTPYTPFTTPXDGGXDWR^54^ by N-terminal labelling and ESI-QTOF mass spectrometry of purified P40 [34]. Cleavage at this site generates an N-terminal fragment of Mpn142 with a predicted mass of 43.9 kDa but the P40 protein migrates with an apparent mass of 36 kDa, a discrepancy of about 9 kDa. Catrein *et al.* [34] hypothesized that further endoproteolytic events removing as much a 9 kDa from the P40 molecule takes place but were unable to accurately determine the location of a cleavage site(s) or confirm the presence of further cleavage fragments of P40. The cleavage event after amino acid 454 creates P40 and P90. Edman degradation of the P90 protein identified the sequence ^455^RAGNSSEDAL^465^ indicating that P90 spans amino acids 455–1218 with a predicted mass of 83.7 kDa [33].

Adhesins are multifunctional proteins that comprise the different functional domains needed to bind to a range of host molecules during the colonisation of host surfaces. Many adhesins are targets of processing events that not only remove signal secretion sequences but also release a range of functional cleavage products. In support of this view, numerous mycoplasma-derived adhesins have shown to be extensively processed [58,59,60,61,62,63,64,65,66,67,68,69,70,71,72] as well as adhesins found in a range of Gram positive [73] and Gram negative pathogens [74,75]. *M. pneumoniae* binds to fibronectin [76]; fibrinogen [77]; plasminogen [78,79]; sialylated receptors [38] and oligosaccharides [80,81]; sulfated glycolipids [82]; laminin, fetuin, and human chorionic gonadotropin [83] but the identities of the proteins localised to the attachment organelle that target these molecules are largely unknown. While most studies have focussed on characterising interactions between P40 and P90 with other proteins in the attachment organelle, their presence on the surface of *M. pneumoniae* in close association with P1 indicates that they may also have roles in adherence to host molecules. To investigate this, we applied systems-wide, affinity chromatography methodologies using fibronectin, actin, heparin, plasminogen, fetuin and surface-exposed proteins from A549 cells as bait to determine whether P40 and P90 may play a role in binding key host molecules. We not only recovered P40 and P90 but also found strong evidence that P90 and P40 are subject to further processing events. To determine precise cleavage sites in P90 and P40, we dimethyl-labelled whole cell lysates of *M. pneumoniae* and characterised neo-N-termini in P90 and P40 by Liquid Chromatography Tandem Mass Spectrometry (LC-MS/MS). These studies provide new insights in the structural and functional capabilities of P90 and P40 and identified regions within these two molecules worthy of further study.

## 2. Experimental Section

### 2.1. Strains and Cultures and Reagents

The M129 *M. pneumoniae* strain was cultured in modified Hayflick’s medium at 37 °C in tissue culture flasks as described previously [84]. Modified Hayflick’s medium contained 21 g PPL broth base without crystal violet, 5 g of d-glucose, 4 mL of 0.5% phenol red, 100 mL of liquid yeast extract (150 g/L), 200 mL heat-inactivated horse serum (56 °C, 30 min) supplemented with 1 g ampicillin (Sigma, A5354, St. Louis, MO, USA) per litre.

Carcinoma lung epithelial (A549) cells were cultured in RPMI 1640 medium (Invitrogen, Grand Island, NY, USA) supplemented with 10% heat inactivated fetal bovine serum at 37 °C with 5% CO_2_ in tissue culture flasks.

Purified fibronectin (Code: 341635) and plasminogen (Code: 528175) from human plasma was supplied by Merck Millipore (Bayswater, Australia). Bovine actin (Code: A3653) and fetuin (Code: F3004) was supplied by Sigma.

### 2.2. Enrichment of *M. pneumoniae* Surface Proteins

#### 2.2.1. Biotinylation

Biotinylation of the *M. pneumoniae* cell surface was carried out using a modification of a protocol described previously [63]. In brief, biotinylation using EZ-link sulfo-NHS-biotin (Thermo Fisher Scientific, Waltham, MA, USA) was added to adherent *M. pneumoniae* cells in culture flasks and performed for 30 s on ice to minimise cell lysis after extensive washing of adherent cells with PBS to remove media components. The reaction was quenched with a final concentration 50 mM Tris-HCl and cells were lysed with 7 M urea, 2 M thiourea, 40 mM Tris-HCl (pH 8.8), 1% (*w*/*v*) C7bZ0. Biotinylated surface proteins were purified by avidin column chromatography and confirmed to be biotinylated by Western blotting using Extravidin-HRP (Sigma).

#### 2.2.2. Trypsin Shaving

Trypsin shaving of *M. pneumoniae* cells was carried out as described previously [61]. In brief, adherent *M. pneumoniae* cells were extensively washed with PBS and then incubated with trypsin from porcine pancreas (Sigma, 50 μg·mL^−1^) for 5 minutes at 37 °C to release surface exposed peptides. Peptides were collected, digested a second time with trypsin Gold MS grade (Promega, Madison, WI, USA) and analysed by LC-MS/MS.

### 2.3. Preparation of M. pneumoniae Whole Cell Lysates for One- and Two-Dimensional Gel Electrophoresis

*M. pneumoniae* whole cell lysates were prepared as previously described [61]. In brief, *M. pneumoniae* cells were extensively washed with PBS and lysed in 7 M urea, 2 M thiourea, 40 mM Tris-HCl, 1% (*w*/*v*) C7BzO detergent (Sigma), followed by three 30 s rounds of sonication at 60% power on ice. Proteins were reduced and alkylated with 5 mM tributylphosphine and 20 mM acrylamide monomers for 90 min at room temperature. Insoluble material was removed by centrifugation and five volumes of acetone added to precipitate protein. After centrifugation, the protein pellet was solubilized in 7 M urea, 2 M thiourea, 1% (*w*/*v*) C7BzO for one- and two-dimensional gel electrophoresis.

### 2.4. One- and Two-dimensional Polyacrylamide Gel Electrophoresis (PAGE)

Protein separation was performed as described in [65,68].

#### 2.4.1. 1D SDS-PAGE

Eighty micrograms of protein was separated on 4%–20% Criterion™ TGX™ Gels (Bio-Rad, Hercules, CA, USA) in Tris-Glycine-SDS buffer (Bio-Rad), fixed and visualized by staining with either Flamingo fluorescent gel stain (Bio-Rad) or Coomassie Blue G-250.

#### 2.4.2. 2D SDS-PAGE

Two hundred-fifty micrograms of protein was cup-loaded onto 11 cm pH 4–7 IPG strips (Bio-Rad) or 6–11 Immobiline drystrips (GE Healthcare, Little Chalfont, UK) rehydrated with 7 M urea, 2 M thiourea, 1% (*w*/*v*) C7BzO. Focusing was performed in a Bio-Rad Protean isoelectric focusing (IEF) cell unit. Following IEF, the strips were equilibrated for 20 min with equilibration (2% SDS, 6 M urea, 250 mM Tris-HCl pH 8.5, 0.0025% (*w*/*v*) bromophenol blue) solution before running in the second-dimension SDS-Polyacrylamide gel electrophoresis (SDS-PAGE).

#### 2.4.3. Trypsin Digest

In-gel trypsin digestion was performed as described in [63]. In brief, gel pieces were destained, dehydrated and incubated with trypsin Gold MS grade (Promega) in 100 mM NH_4_HCO_3_ at 37 °C overnight. Tryptic peptides were then analysed by LC-MS/MS. If necessary, gel pieces were reduced and alkylated with 5 mM TBP, 20 mM acrylamide in 100 mM NH_4_HCO_3_, destained and dehydrated a second time before the addition of trypsin.

### 2.5. Heparin Affinity Chromatography

Affinity purification of *M. pneumoniae* heparin-binding proteins was performed as described in Raymond *et al*. [68] with slight modification. In brief, *M. pneumoniae* cells were extensively washed with PBS and lysed in 10 mM sodium phosphate, 0.1% Triton TX-100, pH 7 with sonication. After centrifugation, ~300 μg of soluble protein was added into an autosampler vial on a Waters 2690 Alliance LC separations module and loaded at 0.5 mL·min^−1^ onto a 1 mL HiTrap Heparin HP column (GE Healthcare) in binding buffer (10 mM sodium phosphate, pH 7). Non-binding proteins were removed with binding buffer. Heparin binding proteins were eluted with an increasing gradient of elution buffer (10 mM sodium phosphate, 2 M sodium chloride, pH 7). Fractionated proteins were separated by 1D-SDS PAGE, in-gel digested with trypsin and analysed by LC-MS/MS.

### 2.6. Identification of Host Binding Proteins (Fibronectin, Plasminogen, Actin and Fetuin)

Avidin purification of *M. pneumoniae* host molecule binding proteins was performed as described in Raymond *et al*. [70] with slight modifications. In brief, 1 mg of purified host protein (either fibronectin, plasminogen, actin or fetuin) was biotinylated and bound to avidin agarose. The avidin beads were incubated for 16 h at 4 °C with *M. pneumoniae* cells lysed with 1% (*w*/*v*) C7BzO (Sigma) in PBS (pH 7.8). Non-binding proteins were washed with PBS and host binding proteins were eluted with 7 M urea, 2 M thiourea, 40 mM Tris-HCl and 1% (*w*/*v*) C7BzO. Eluants were pooled, concentrated via acetone precipitation for separation by 1D-SDS PAGE, in-gel digested with trypsin and analysed by LC-MS/MS.

### 2.7. Identification of A549 Binding Proteins

Avidin purification of *M. pneumoniae* proteins that bind A549 surface proteins was performed as described in Raymond *et al*. [68] with modifications. In brief, A549 cells were grown to ~80% confluency and biotinylated in the flask after extensive washing with PBS. After quenching excess biotin with a final concentration of 50 mM Tris-HCl, A549 cells were lysed in 1% (*w*/*v*) C7BzO (Sigma) in PBS (pH 7.8) with sonication. A549 whole cell lysate was added to avidin agarose beads following centrifugation. The beads were washed four times (5 mL per wash) with PBS to remove non-biotinylated proteins. The beads were then incubated for 16 h at 4 °C with *M. pneumoniae* cells lysed with 1% (*w*/*v*) C7BzO (Sigma) in PBS (pH 7.8). Non-binding *M. pneumoniae* proteins were removed by washing four times again (5 mL per wash) with PBS and A549 binding proteins were eluted with 7 M urea, 2 M thiourea, 40 mM Tris-HCl and 1% (*w*/*v*) C7BzO (elution 1). Biotinylated surface A549 proteins that bound strongly to avidin-agarose were eluted with 30% acetonitrile and 0.4% trifluoroacetic acid (elution 2). Each of the eluents were concentrated via acetone precipitation for separation by 1D-SDS PAGE, in-gel digested with trypsin and analysed by LC-MS/MS.

### 2.8. Liquid Chromatography Tandem Mass Spectrometry (L*C*-MS/MS)

LC-MS/MS was performed as described in Raymond *et al*. [68]. In brief, 15 μL of sample containing up to 5 μg of protein was loaded into an autosampler vial in an Eksigent AS-1 autosampler connected to a Tempo nanoLC system (Eksigent, Livermore, CA, USA) with a C8 Cap Trap column (Michrom Biosciences, Auburn, CA, USA). The peptides were washed onto a PicoFrit column (75 μm × 150 mm) packed with Magic C18AQ resin (Michrom Biosciences, CA). Peptides were eluted from the column into the source of a QSTAR Elite hybrid quadrupole-time-of-flight mass spectrometer (Sciex, Redwood, CA, USA). Eluted peptides were ionized from the PicoFrit at 2300 V. An Intelligent Data Acquisition (IDA) experiment was performed, with a mass range of 350–1500 Da continuously scanned for peptides of charge state 2+ to 5+ with an intensity of more than 30 counts/scan. Selected peptides were fragmented and the product ion fragment masses were measured over a mass range of 50–1500 Da.

### 2.9. MS/MS Data Analysis

Mascot (Matrix Science, London, UK, version 6.1) was used to search MS/MS data files as previously described Raymond *et al*. [68] with modifications. In brief, files were searched against the MSPnr100 database [85] with the following parameters. Fixed modifications: none. Variable modifications: propionamide, oxidized methionine, deamidation. Enzyme: semi-trypsin. Number of allowed missed cleavages: 3. Peptide mass tolerance: 100 ppm. MS/MS mass tolerance: 0.2 Da. Charge state: 2+, 3+ and 4+. For samples collected from the “Biotinylation enrichment of surface proteins” and “Avidin purification of A549 interacting proteins”, variable modifications also included NHS-LC-Biotin (K) and NHS-LC-Biotin (N-term). “Avidin purification of A549 interacting proteins” was also searched against *homo sapiens* entries in MSPnr100 to identify biotinylated surface A549 proteins.

### 2.10. Dimethyl Labelling of M. pneumoniae Proteins

Protein labelling was performed on 1 mg of *M. pneumoniae* protein by the addition of 40 mM formaldehyde (ultrapure grade) (Polysciences, Warrington, PA, USA) in the presence of 20 mM sodium cyanoborohydride, buffered with 100 mM HEPES solution adjusted to pH 6.7 in a final volume of 1 mL, and incubated at 37 °C for a minimum of 4 h. The reaction was quenched by the addition of 100 mM ammonium bicarbonate and precipitated with 8 volumes of acetone and 1 volume of methanol at −80 °C for 3 h. The precipitated protein was then pelleted by centrifugation at 14,000 g and washed with 5 volumes of methanol. The protein pellet was resuspended in 50 mM sodium hydroxide, pH 8.0 and digested with trypsin for 16 h at 37 °C prior to clean up by SiliaPrepX™ HLB Polymeric SPE cartridges (Silicycle, Québec City, QC, Canada) and analysis by LC–MS/MS (Sciex 5600 and Thermo Scientific Q Exactive™, Waltham, MA, USA ).

### 2.11. Liquid Chromatography Tandem Mass Spectrometry (L*C*-MS/MS): Sciex 5600

Peptides from dimethyl labelled *M. pneumoniae* protein were separated by nanoLC using an Ultimate nanoRSLC UPLC and autosampler system (Dionex, Amsterdam, The Netherlands). Samples (2.5 μL) were concentrated and desalted onto a micro C18 precolumn (300 μm × 5 mm, Dionex) with H_2_O:CH_3_CN (98:2, 0.1% TFA) at 15 μL/min. After a 4 min wash, the pre-column was switched (Valco 10 port UPLC valve, Valco, Houston, TX, USA) into line with a fritless nano column (75 μm × ~15 cm) containing C18AQ media (1.9 μ, 120 Å Dr Maisch, Ammerbuch-Entringen, Germany). Peptides were eluted using a linear gradient of H_2_O:CH_3_CN (98:2, 0.1% formic acid) to H_2_O:CH_3_CN (64:36, 0.1% formic acid) at 200 nL/min over 240 min. High voltage 2000 V was applied to low volume Titanium union (Valco, Houston, TX, USA) with the tip positioned ~0.5 cm from the curtain plate (T = 150 °C) of a 5600^+^ mass spectrometer (Sciex, Toronto, ON, Canada). Positive ions were generated by electrospray and the 5600^+^ operated in information dependent acquisition mode (IDA).

A survey scan *m*/*z* 350–1750 was acquired (PWHH resolution ~30,000, 0.25 s acquisition time) with autocalibration enabled (at ~6 h intervals). Up to the 10 most abundant ions (>300 counts) with charge states >+2 and <+5 were sequentially isolated (width *m*/*z* ~3) and fragmented by CID with an optimal CE chosen based on *m*/*z* (product ion spectra were acquired at a resolution ~20,000 PWHH in 0.15 s). *m*/*z* ratios selected for MS/MS were dynamically excluded for 30 or 45 s.

Peak lists were generated using Mascot Daemon/Mascot Distiller (Matrix Science) or ProteinPilot (Sciex, v4.5) using default parameters, and submitted to the database search program Mascot (version 2.5.1, Matrix Science). Search parameters were: Precursor tolerance 10 ppm and product ion tolerances ± 0.05 Da; oxidation (M), deamidation (NQ), propionamide (C), Dimethyl (K), Dimethyl (N-term) specified as variable modifications; enzyme specificity was semi-ArgC; 1 missed cleavage was possible and the non-redundant protein database from NCBI (National Center for Biotechnology Information, January 2015) searched.

### 2.12. Liquid Chromatography Tandem Mass Spectrometry (L*C*-MS/MS): Thermo Scientific Q Exactive™

Peptides from dimethyl labelled *M. pneumoniae* protein were separated by nanoLC using an Ultimate nanoRSLC UPLC and autosampler system (Dionex, Amsterdam, The Netherlands). Samples (2.5 μL) were concentrated and desalted onto a micro C18 precolumn (300 μm × 5 mm, Dionex) with H_2_O:CH_3_CN (98:2, 0.1% TFA) at 15 μL/min. After a 4 min wash, the pre-column was switched (Valco 10 port UPLC valve, Valco, Houston, TX, USA) into line with a fritless nano column (75 μm × ~35 cm) containing C18AQ media (1.9 μ, 120 Å Dr Maisch, Ammerbuch-Entringen Germany). Peptides were eluted using a linear gradient of H_2_O:CH_3_CN (98:2, 0.1% formic acid) to H_2_O:CH_3_CN (64:36, 0.1% formic acid) at 200 nL/min over 30 or 240 min. High voltage 2000 V was applied to low volume Titanium union (Valco) with the column oven heated to 45 °C (Sonation, Biberach, Germany) and the tip positioned ~0.5 cm from the heated capillary (T = 300 °C) of a QExactive Plus mass spectrometer (Thermo Scientific). Positive ions were generated by electrospray and the QExactive operated in data dependent acquisition mode (DDA).

A survey scan *m*/*z* 350–1750 was acquired (resolution = 70,000 at *m*/*z* 200, with an AGC target value of 10^6^ ions) and lockmass was enabled (*m*/*z* 445.12003) Up to the 10 most abundant ions (>80,000 counts, underfill ratio 10%) with charge states >+2 and <+7 were sequentially isolated (width *m*/*z* 2.5) and fragmented by HCD (NCE = 30) with an AGC target of 10^5^ ions (resolution = 17,500 at *m*/*z* 200). *m*/*z* ratios selected for MS/MS were dynamically excluded for 30 or 45 s.

Peak lists were generated using Mascot Daemon/Mascot Distiller (Matrix Science) or Proteome Discoverer (Thermo Scientific, v1.4) using default parameters, and submitted to the database search program Mascot (version 2.5.1, Matrix Science). Search parameters were: Precursor tolerance 4 ppm and product ion tolerances ± 0.05 Da; oxidation (M), deamidation (NQ), propionamide (C), Dimethyl (K), Dimethyl (N-term) specified as variable modifications; enzyme specificity was semi-ArgC; one missed cleavage was possible and the non-redundant protein database from NCBI (January 2015) searched.

### 2.13. Bioinformatic Analysis of Mpn142

Bioinformatic analysis of Mpn142 used online resources: ProtParam [86], Clustal Omega [87], TMpred [88], COILS (Addition of “yes” to 2.5 fold weighting of positions a,d) [89] and PONDR^®^ (VSL2 and VL3 predictors) [90,91]. Using ScanProsite [92], the (X-[HRK]-[HRK]-X-[HRK]-X) motif identified by Cardin & Weintraub (1989) [93] was used to predict putative heparin binding motifs and the (X-[HRK]-X-[HRK]-[HRK]-X) motif is implicated in putative heparin sulphate binding sites [94].

## 3. Results

### 3.1. Defining P40 and P90 on the Surface of M. pneumoniae

**Figure 1:**
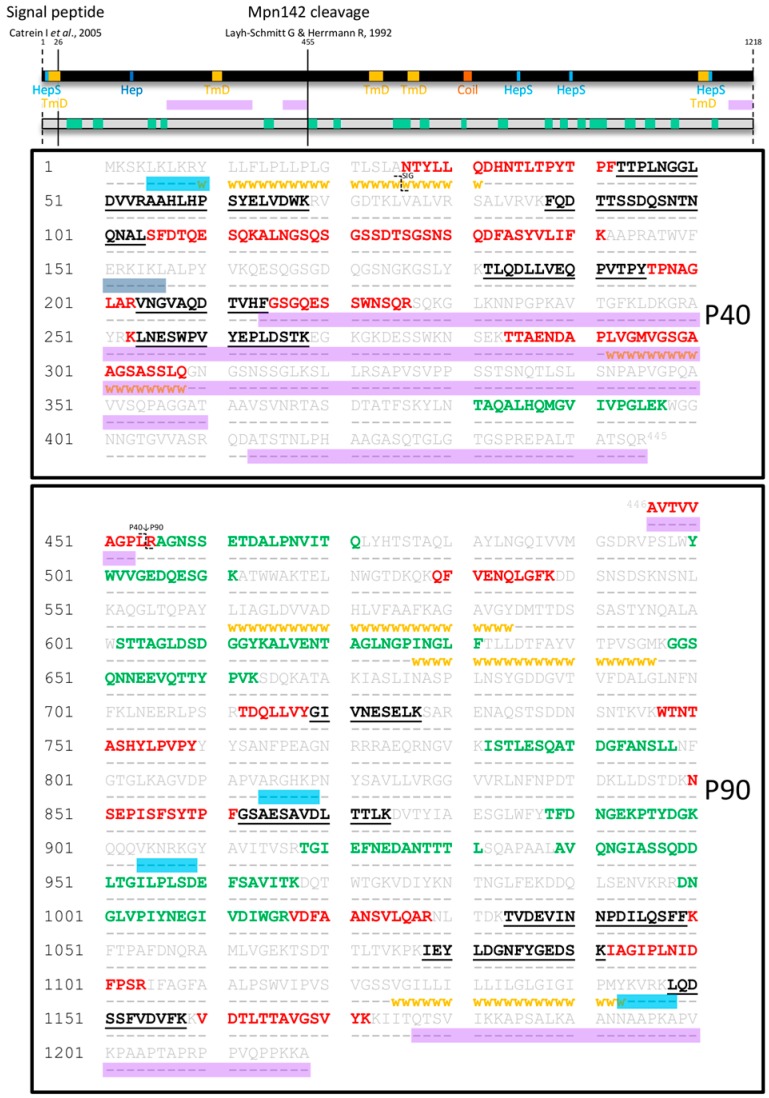
Peptides identified in surface proteome analysis of Mpn142. Full length Mpn142 is represented as a black bar and its amino acid sequence (grey) is shown underneath. Tryptic peptides identified by shaving the surface of *M. pneumoniae* are coloured green while tryptic peptides derived from a 2D gel loaded with biotinylated (surface) proteins of *M. pneumoniae* are coloured red. Tryptic peptides that were common to both surface analyses are coloured black and underlined. Bioinformatics tools were used to predict coiled-coils (orange box; COILS), transmembrane domains (yellow squares/**ⱳⱳⱳⱳⱳ**; TMpred), disordered regions (purple boxes; PONDR^®^: VSL2 and VSL3 predictor), heparin-binding motifs (dark blue box; ScanProsite with “*X-[HRK]-[HRK]-X-[HRK]-X*” motif) and motifs implicated in heparan sulfate binding (light blue boxes; ScanProsite with “*X-[HRK]-X-[HRK]-[HRK]-X*” motif). The two previously reported cleavage sites after amino acid 25 and 454 are indicated as unbroken lines across the black bar and with the symbol **ʅ** in the sequence.

Surface accessible proteins labelled with biotin were recovered by streptavidin chromatography and separated by 2D-PAGE. All protein spots were cut from the gel, digested with trypsin and analysed by LC-MS/MS as described previously [65,68]. Tryptic peptides that mapped to Mpn142 were identified in two spot trains with masses of approximately 37 kDa and 84 kDa (data not shown). Twenty seven tryptic peptides (Mascot scores >50) that mapped to the 40 kDa protein spanned amino acids 26–308 of the P40 molecule (Figure 1). The first peptide ^26^NTYLLQDHNTLTPYTPFTTPLNGGLDVVR^54^ was semi-tryptic in composition and represented the mature N-terminus of P40 after removal of the leader peptide (Table 1). A novel cleavage site with the sequence ^364^NRT↓ASD^371^ defines the largest fragment we identified in the N-terminal half of Mpn142 and delineates the *C*-terminus of P40 (Fragment 3). Dimethyl-labelling experiments identified semi-tryptic peptides that confirmed that cleavage occurs in multiple sites in this region of Mpn142 suggesting that it is readily accessible to proteases or is further processed after the initial cleavage event that creates P40 (Table 1). Fragment 3 spans amino acids 26–368 with a predicted mass of 36.3 kDa (pI = 9.28) and was identified in affinity columns loaded with heparin and fibronectin at masses consistent with this predicted size of the molecule. A putative heparin-binding domain with the sequence ^151^ERKIKL^156^ was identified within the P40 sequence consistent with our ability to recover Fragment 3 from heparin-agarose. Further studies are needed to confirm interactions between Fragment 3 with heparin and fibronectin.

**Table 1:** N-terminal dimethyl labelled and semi-tryptic peptides identified in Mpn142.

#	Peptide Sequence	Score	E-Value	5600	QE
	**N-terminal Dimethyl Labelled**				
N1	-.^**1**^**M**KSKLKLKR^9^.Y	11 *	0.72	1	1
N2	L.^**25**^**A**NTYLLQDHNTLTPYTPFTTPLNGGLDVVR^54^.A	36	0.0024	1	3
N3	A.^**26**^**N**TYLLQDHNTLTPYTPFTTPLNGGLDVVR^54^.A	142	3.5 × 10^−13^	4	4
N4	R.^**153**^**K**IKLALPYVKQESQGSGDQGSNGKGSLYKTLQDLLVEQPVTPYTPNAGLARVNGV^207^.A	66	2.3 × 10^−5^	-	1
N5	R.^**368**^**T**ASDTATFSK^377^.Y	50	0.00068	-	1
N6	R.^**368**^**T**ASDTATFSKYLNTAQALHQMGVIVPGLEKWGGNNGTGVVAS^409^.R	55	1.2 × 10^−5^	2	-
N7	R.^**368**^**T**ASDTATFSKYLNTAQALHQMGVIVPGLEKWGGNNGTGVVASR^410^.Q	146	1.1 × 10^−13^	2	3
N8	R.^**368**^**T**ASDTATFSKYLNTAQALHQMGVIVPGLEKWGGNNGTGVVASRQ^411^.D	148	5.2 × 10^−14^	1	4
N9	R.^**368**^**T**ASDTATFSKYLNTAQALHQMGVIVPGLEKWGGNNGTGVVASRQD^412^.A	163	1.2 × 10^−15^	-	2
N10	R.^**368**^**T**ASDTATFSKYLNTAQALHQMGVIVPGLEKWGGNNGTGVVASRQDA^413^.T	80	9.8 × 10^−8^	-	2
N11	R.^**368**^**T**ASDTATFSKYLNTAQALHQMGVIVPGLEKWGGNNGTGVVASRQDAT^414^.S	62	3.9 × 10^−6^	-	2
N12	T.^**369**^**A**SDTATFSKYLNTAQALHQMGVIVPGLEKWGGNNGTGVVASR^410^.Q	138	2.7 × 10^−13^	2	4
N13	R.^**446**^**A**VTVVAGPLR^455^.A	72	1.2 × 10^−5^	3	4
N14	A.^**447**^**V**TVVAGPLR^455^.A	48	0.0024	3	4
N15	L.^**696**^**G**LNFNFKLNEER^707^.L	13 *	1.3	2	2
N16	R.^**731**^**E**NAQSTSDDNSNTKVKWTNTASHYLPVPYYYSANFPEAGNRR^772^.R	21 *	0.015	-	1
N17	A.^**775**^**E**QRNGVKISTLESQATDGFANSLLNFGTGLKAGVDPAPVAR^815^.G	41	0.00025	-	1
N18	A.^**1022**^**N**SVLQARNLTDKTVDEVINNPDILQSFFKFTPAFDNQR^1059^.A	84	8.8 × 10^−7^	3	1
N19	K.^**1190**^**A**ANNAAPKAPVKPAAPTAPRPPVQPPKKA^1218^.-	59	4.7 × 10^−6^	-	1
N20	A.^**1191**^**A**NNAAPKAPVKPAAPTAPRPPVQPPKKA^1218^.-	99	1.7 × 10^−9^	1	3
N21	A.^**1192**^**N**NAAPKAPVKPAAPTAPRPPVQPPKKA^1218^.-	41	0.00014	-	1
N22	N.^**1194**^**A**APKAPVKPAAPTAPRPPVQPPKKA^1218^.-	44	0.00019	-	2
N23	A.^**1195**^**A**PKAPVKPAAPTAPRPPVQPPKKA^1218^.-	18 *	0.051	-	1
N24	P.^**1197**^**K**APVKPAAPTAPRPPVQPPKKA^1218^.-	21 *	0.029	-	1
	**Semi-tryptic Truncated C-terminal Peptides**				
S1	R.^253^KLNESWPVYEPLDSTKEGKGKDESSWKNSEKTTAENDAPLVGMVGSGA**A**^**301**^.G	73	5.4 × 10^−7^	1	-
S2	R.^253^KLNESWPVYEPLDSTKEGKGKDESSWKNSEKTTAENDAPLVGMVGSGAAG**S**^**303**^.A	87	1.4 × 10^−8^	1	-
S3	R.^253^KLNESWPVYEPLDSTKEGKGKDESSWKNSEKTTAENDAPLVGMVGSGAAGS**A**^**304**^.S	30 *	0.0071	1	-
S4	R.^253^KLNESWPVYEPLDSTKEGKGKDESSWKNSEKTTAENDAPLVGMVGSGAAGSA**S**^**305**^.S	40	0.00072	1	-
S5	R.^253^KLNESWPVYEPLDSTKEGKGKDESSWKNSEKTTAENDAPLVGMVGSGAAGSAS**S**^**306**^.L	39	0.00077	-	1
S6	R.^253^KLNESWPVYEPLDSTKEGKGKDESSWKNSEKTTAENDAPLVGMVGSGAAGSASSL**Q**^**308**^.G	86	1.4 × 10^−7^	1	-
S7	R.^1029^NLTDKTVDEVINNPDILQSFFKFTPAFDNQRAML**V**^**1063**^.G	45	0.0023	-	1
	**Semi-tryptic Truncated N-terminal Peptides**				
S8	D.^**810**^**P**APVARGHKPNYSAVLLVR^828^.G	65	2.3 × 10^−6^	-	1
S9	N.^**983**^**G**LFEKDDQLSENVKRR^998^.D	26 *	0.033	-	1
S10	N.^**1193**^**N**AAPKAPVKPAAPTAPRPPVQPPKKA^1218^.-	25 *	0.015	-	1
	**P1 adhesin Cleaved Peptides**				
C1	R.^1594^LKQTSAAKP**G**^**1603**^.A (Semi-tryptic)	24 *	0.045	-	1
C2	T.^**1598**^**S**AAKPGAPRPPVPPKPGAPKPPVQPPKKPA^1627^.- (Dimethyl labeled)	58	4.5 × 10^−6^	-	2

All peptides have a score >32 and an E-value < 0.05 unless indicated by * which signifies that the peptide was either identified over several replicates or correlates with predicted fragments in this study. # indicates number. Cleavage sites are located beside the bold underlined amino acid (left for N-terminus and right for C-terminus). Amino acid number is written as superscript at the start and end of the peptide. Highest ion score and lowest E-value for the peptide identified across the replicates is listed. The last two columns contain the number of times the peptide was identified by either the Sciex 5600 TripleTOF or Thermo Q Exactive Plus out of a total of six biological replicates. The last two peptides are C-terminal cleavage sites identified for P1 (Uniprot #: P11311).

**Figure 2:**
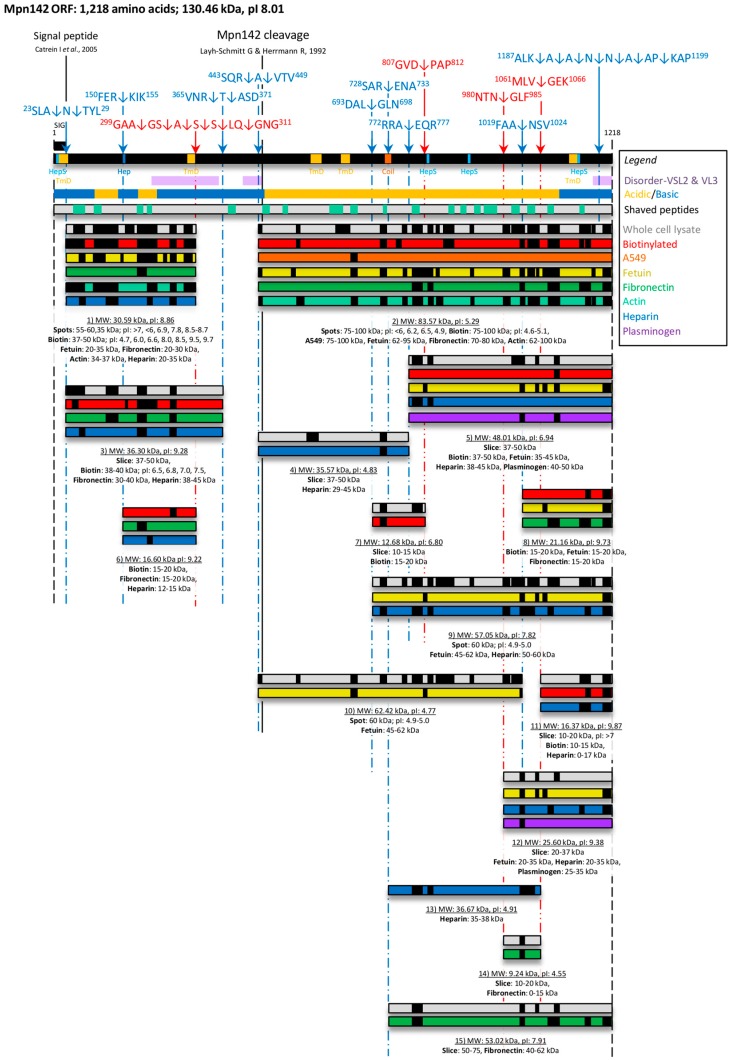
Cleavage map of Mpn142. Full length Mpn142 is represented as a black bar and cleavage products shown underneath. Cleavage sites were determined by mapping neo-N-termini generated by N-terminal dimethyl labelling (blue broken lines) and identifying semi-tryptic peptides (red broken lines). Cleavage sites are indicated by the arrows and peptide sequences shown above the bar. Putative glycosaminoglycan-binding sites (HepS/Hep), transmembrane domains (TmD), coiled-coils (Coil) and disordered regions identified using ScanProsite, TMpred, COILS and PONDR^®^, respectively are depicted. Regions within Mpn142 that are enriched in acidic amino acids D/E (**yellow**) and basic amino acids K/R/H (**blue**) are shown beneath the black bar representing Mpn142. Shaved peptides are depicted by the green boxes within the grey bar. Peptides spanning fragments of Mpn142 obtained from 1D/2D SDS PAGE of whole cell lysates (**grey** bars); recovery of biotinyled proteins (**red** bars); or A549 (**orange** bar), fetuin (**yellow** bars), fibronectin (**green** bars), actin (**teal** bars), heparin (**dark blue** bars) or plasminogen (purple bars) based affinity chromatography were identified by LC-MS/MS (small black boxes). Beneath each fragment is the assigned fragment number, predicted theoretical mass and pI predicted by ProtParam (underlined). All fragments were identified in a defined mass range. The signal peptide and previously determined Mpn142 cleavage sites are presented as unbroken black lines.

Twelve tryptic peptides (Mascot scores > 50) spanning amino acids 446–1172 were mapped to P90 (Fragment 2). Notably, the first peptide ^446^AVTVVAGPLR^455^ is semi-tryptic in composition and is the first peptide within P90 (Figure 1). The most C-terminal peptide in P90 has the sequence ^1160^VDTLTTAVGSVYK^1172^. Tryptic peptides that mapped to a C-terminal 90 kDa fragment of Mpn142 were isolated by affinity chromatography using fetuin, fibronectin, actin and A549 surface proteins as bait indicating that P90 may bind to fibronectin, actin, fetuin and other undefined receptors on the surface of A549 cells (Figure 2).

Tryptic peptides spanning Mpn142 that were released during mild trypsin hydrolysis of *M. pneumoniae* cells were identified by LC-MS/MS. Twenty one peptides spanning Mpn142 including six peptides between amino acids 43–397 in P40 and fifteen peptides spanning amino acids 456–1158 in P90 were identified (see green boxes in the grey bar in Figure 2). The peptide ^381^TAQALHQMGVIVPGLEK^397^ is flanked on either side by predicted disordered regions and is the only peptide that mapped to this region, suggesting that it is a preferential site for proteolysis in Mpn142. These data confirm that most of Mpn142 is exposed on the surface of *M. pneumoniae*.

### 3.2. Bioinformatic Analysis of Mpn142

**Figure 3:**
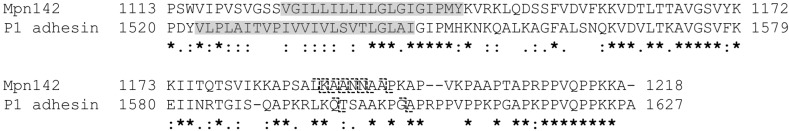
Alignment of the C-terminal sequence of Mpn142 and P1**.** A Clustal Omega alignment of the amino acid sequence spanning 1113–1218 of Mpn142 against the sequence spanning 1520–1627 of P1. Predicted transmembrane domains are highlighted in grey (TMpred score 2518) for Mpn142 and predicted by Nakane *et al.* (2010) using the SMART algorithm [37]. Cleavage sites in the sequence are denoted by the symbol ʅ. * indicate conserved residues: indicate similar amino acids and indicate weakly similar amino acids.

The online PONDR^®^ tool (VSL2 and VL3 predictors) [90,91] identified three major disordered regions spanning more than 40 consecutive amino acids from amino acid 214–360 and 413–453 which aligns with C-terminal half of P40 (Figure 1). The third disordered region resides in the C-terminal region of P90 between amino acids 1178–1218 and spanned an unusual sequence of amino acids enriched in alanine, proline and lysine residues located at the *C*-terminus of the molecule (Figure 3). Interestingly, a similar run of amino acids was identified in the *C*-terminus of the P1 adhesin derived from the *mpn141* gene found in the same polycistronic unit (Figure 3). Notably, the disordered region spanning amino acids 413–453 encompasses the two consecutive cleavage sites ^443^SQR↓A↓VTV^449^ that separates P40 from P90 while the longest disordered region from 214 to 360 encompasses cleavage sites ^299^GAA↓GS↓A↓S↓S↓LQ↓^308^ that defines the *C*-terminus of P40. Evidence to support these cleavage events is derived from the identification of dimethyl labelled semi-tryptic peptides (neo-N-termini) by LC-MS/MS (see Table 1). We rarely found tryptic peptides that mapped to amino acids 310–440. We also identified a putative heparin binding site with the sequence ^151^ERKIKL^156^ and a series of regions which may be implicated in heparan sulfate binding domains with the sequences: ^151^ERKIKL^156^, ^5^LKLKRY^10^, ^814^ARGHKP^819^, ^904^VKNRKG^909^ and ^1143^YKVRKL^1148^ in Mpn142 prompting us to use heparin as bait in affinity chromatography experiments to enrich for cleavage fragments derived from Mpn142.

### 3.3. Cleavage Fragments in the N-terminus of Mpn142

Dimethyl labelling of neo-N-termini provided evidence that Mpn142 is subject to considerable posttranslational processing. The list of dimethyl labelled peptides identified by LC-MS/MS is shown in Table 1. To find evidence that these cleavage events generated functionally important cleavage fragments of Mpn142, we characterised tryptic peptides derived from size-fractionated whole cell lysates of *M. pneumoniae* strain M129 separated by SDS-PAGE and proteins captured by affinity chromatography loaded with different bait including actin, fetuin, plasminogen, heparin, fibronectin and surface proteins from A549 cells (Figure 2 and Figure S1). In addition, we identified fragments of Mpn142 from 2D SDS-PAGE gels loaded separately with M129 whole cell lysates, and biotinylated surface proteins, and characterised them by LC-MS/MS (Supplementary File). Using these combined approaches, we identified 15 fragments of Mpn142 (Figure 2, Supplementary File). Most of the fragments were identified at the predicted mass and pI calculated by ProtParam (Table S1). Blast analysis of each of the fragments is shown in Table S2. Consistent with data described above, we were unable to find a cleavage fragment spanning amino acids 26–454 with a predicted mass of 44.9 kDa which represents the largest possible N-terminal cleavage fragment of Mpn142. Fragments 1, 3, and 6 span different regions of the N-terminus of Mpn142. Fragment 1 has a predicted mass of 30.6 kDa (pI = 8.86), spans amino acids 26–308 and was identified in a series of gel spots at 35 kDa (pI ranging from 6.9–8.7) from 2D gels loaded with whole cell lysates, and biotinylated surface proteins of *M. pneumoniae*, and from affinity columns loaded with fetuin, actin, heparin and fibronectin. These data suggest that Fragment 1 binds actin, fetuin, heparin and fibronectin and is present on the surface of *M. pneumoniae*. Fragment 6 spans amino acid 153–308 with a predicted mass of 16.6 kDa and was also recovered from gel slices in the range of 15–20 kDa containing biotinylated surface proteins, *M. pneumoniae* proteins eluted during affinity chromatography using fibronectin as bait, and a SDS-polyacrylamide gel slice (12–15 kDa) containing proteins that eluted from a heparin-agarose column. These data suggest that Fragment 6 binds heparin and fibronectin and is present on the surface of *M. pneumoniae*.

### 3.4. Cleavage Fragments Residing in the *C*-terminus of Mpn142

Several dimethylated N-terminal peptides were identified in the C-terminal region of Mpn142 (Table 1). Four cleavage sites reside between amino acids 695–810 and three cleavage sites were identified between amino acids 982–1063. In total, we identified 12 fragments spanning different regions of the C-terminal two thirds of Mpn142.

Fragment 5 is predicted to start at position 775 (see dimethyl peptide N17 in Table 1) and terminates at amino acid 1218, generating a protein with a predicted mass of 48 kDa. Fragment 5 was recovered from affinity columns loaded with plasminogen (two peptides; gel slice 40–50 kDa), fetuin (six peptides; gel slice from 35 to 45 kDa), heparin (two peptides; gel slice 38–45 kDa) and from a gel slice (37–50 kDa) containing size-fractionated *M. pneumoniae* proteins (six peptides). Peptide coverage from each affinity chromatography experiment is depicted in Figure 2.

Fragment 4 (limited evidence for its existence in this study) is predicted to span amino acids 446–774 and has a predicted mass of 35.6 kDa. It was identified (two tryptic peptides) from a gel slice (37–50 kDa) containing size-fractionated *M. pneumoniae* proteins and from size fractionated proteins (29–45 kDa) eluted during heparin agarose chromatography.

Fragment 7 spans amino acids 696–809, has a predicted mass of 12.7 kDa and was recovered from a gel slice (10–15 kDa) containing size-fractionated *M. pneumoniae* proteins (two tryptic peptides). It is predicted to be derived from a cleavage event at position 696 (^693^DAL↓GLN^698^) and ends at a cleavage event at position 809 in the sequence ^807^GVD↓PAP^812^ (see semi-tryptic peptide S8 and dimethyl peptide N15 in Table 1). We also identified a similar fragment from captured biotinylated surface proteins in a gel slice spanning 15–20 kDa (Figure 2).

Fragment 8 is delineated by a cleavage event at position 1021 within the sequence ^1019^FAA↓NSV^1024^ (see dimethyl peptide N18 in Table 1) at the N-terminus and ends at the *C*-terminus of Mpn142 (position 1218) (Figure 2). It has a predicted mass of 21 kDa and was recovered in three separate experiments in gel slices spanning 15–20 kDa loaded with biotinylated surface proteins of *M. pneumoniae* (two peptides) and affinity columns loaded with fetuin (two peptides) and fibronectin (six peptides) (Figure 2).

Fragments 9 and 10 span different but overlapping regions of P90. Notably, peptide coverage from a protein spot of 60 kDa spanned all of P90 indicating that there are two overlapping fragments co-migrating in the one spot. We predict that Fragment 9 commences at position 696 at the cleavage site ^693^DAL↓GLN^698^ (see dimethyl peptide N15 in Table 1) and spans the C-terminus of Mpn142 generating a theoretical fragment with a mass of 57.1 kDa (p*I* = 7.82). Further support was provided by the identification of nine tryptic peptides mapping to a fragment recovered from heparin-agarose in a gel slice spanning 50–60 kDa. Six tryptic peptides mapping to the C-terminal 90 kDa region of Mpn142 were also identified by LC-MS/MS in a protein recovered from an affinity column loaded with fetuin as bait, in a gel slice with masses ranging from 45 to 62 kDa (Figure 2). As such, our data is consistent with Fragment 10 commencing at the true N-terminus of P90 (see dimethyl peptide N13 in Table 1) at cleavage site ^443^SQR↓AVTV^450^ and spanning the central region of Mpn142, ending at position 1,021 at the cleavage site ^1019^FAA↓NSV^1024^. Fragment 10 has a theoretical mass of 62.4 kDa (pI = 4.77).

Fragment 11 was recovered from SDS-PAGE fractionated *M. pneumoniae* proteins in a gel slice spanning 10–20 kDa. Our data is consistent with Fragment 11 commencing at position 1064 at cleavage site ^1061^MLV↓GEK^1066^ (see semi-tryptic peptide S7 in Table 1) and ending at position 1218. Fragments of a similar size were recovered from heparin agarose in a gel slice containing putative heparin-binding proteins up to 17 kDa in size and from a streptavidin column loaded with biotinylated surface proteins of *M. pneumoniae* with masses between 10 to 15 kDa (Figure 2). Fragment 11 is predicted to be 16.4 kDa.

Fragment 12 comprises tryptic peptides spanning the C-terminal 25.6 kDa of Mpn142 and resides in gel slices from 20 to 37 kDa in size fractionated *M. pneumoniae* proteins recovered from affinity chromatography experiments using fetuin, heparin and plasminogen as bait. Peptide coverage was consistent with Fragment 12 commencing at position 982 in the sequence ^980^NTN↓GLF^985^ and ending at position 1218 of Mpn142, generating a protein with a predicted mass of 25.6 kDa.

Fragment 13 was recovered from a heparin-agarose column and is defined by four tryptic peptides residing in a region of Mpn142 spanning amino acids 774–1063, consistent with cleavage events commencing at position ^772^RRA↓EQR^777^ and ending at position ^1061^MLV↓GEK^1066^ (see dimethyl peptide N17 and semi-tryptic peptide S7 in Table 1). Fragment 13 has a predicted mass of 36.7 kDa and encompasses a region of Mpn142 that displays two putative heparan sulfate-binding domains with the X-[HRK]-X-[HRK]-[HRK]-X motif (^814^ARGHKP^819^ and ^904^VKNRKG^909^).

Evidence for the existence of Fragment 14 is denoted by the identification of two tryptic peptides that map to a small fragment of Mpn142 recovered from gel slices containing *M. pneumoniae* proteins from 10 to 20 kDa and from proteins eluted from an affinity column where fibronectin has been used as bait. Our data is consistent with Fragment 14 commencing at position 982 (^980^NTN↓GLF^985^) and ending at position 1063 (^1061^MLV↓GEK^1066^) generating a fragment with a predicted mass of 9.2 kDa.

Fragment 15 is predicted to spans amino acids 731–1218 (cleavage site ^728^SAR↓ENA^733^ and dimethyl peptide N16 in Table 1) and has a predicted mass of 53 kDa. We identified five tryptic peptides spanning this fragment in a gel slice containing proteins 50–75 kDa and in size fractioned (40–62 kDa) *M. pneumoniae* proteins (eight peptides) eluted from an affinity column loaded with fibronectin (Figure 2).

Notably, we identified a series of cleavage events in the *C*-terminal predicted disorder region from position 1190 that removed one amino acid at a time from the N-terminus of the peptide with sequence ^1190^AANNAAPKAPVKPAAPTAPRPPVQPPKKA^1218^ (Table 1: dimethyl peptides N18–N24 and semi-tryptic peptide S10). These events release a *C*-terminal peptide from Mpn142 that is enriched in alanine/valine (11 residues), lysine/arginine (five residues) and proline (nine residues) residues and is highly similar in sequence to the *C*-terminus of the P1 adhesin (Mpn141) (Figure 3).

## 4. Discussion

A growing body of evidence exists to suggest that molecules that reside on the cell surface of mycoplasmal pathogens are processed into discrete functional domains via a process known as ectodomain shedding [58,59,60,61,62,63,64,65,66,67,68,69,70,71,72]. While the majority of this evidence comes from recent studies of adhesin molecules in *M. hyopneumoniae*, there is ample evidence that surface-accessible proteins in *M. pneumoniae* including Mpn142 and several uncharacterized lipoproteins (Mpn052, Mpn284, Mpn288, Mpn376, Mpn400, Mpn408, Mpn444, Mpn456, Mpn474 and Mpn491) are processed at multiple sites [95]. However, in most instances, precise cleavage events have not been mapped. Previous studies have shown that *M. pneumoniae* has an affinity for a wide variety of host receptors including fibronectin [76]; fibrinogen [77]; plasminogen [78,79]; sialylated receptors and oligosaccharides [38,80,81]; sulfated glycolipids [82]; laminin, fetuin, and human chorionic gonadotropin [83], but the identity of adhesins that bind them have not been characterized in detail. To characterise the processing events and to determine if the products of cleavage are potential adhesins, we developed systems-wide, affinity chromatography methodologies to recover proteins that interact with important host cell surface molecules such as heparin, fibronectin, actin, plasminogen, fetuin and proteins on the surface of A549 cells and identified them by LC-MS/MS. While this approach suggests a direct interaction between *M. pneumoniae* proteins and the bait, definitive proof is lacking because a subset of the captured proteins may interact with proteins that bind directly to the bait. In *M. pneumonia*, pyruvate dehydrogenase β (PdhB) and elongation factor Tu (Ef-Tu) bind fibronectin [76], glyceraldehyde-3-phosphate dehydrogenase binds fibrinogen [77] and PdhA, PdhB and PdhC subunits bind plasminogen [78,79] on the surface. However, it is not clear how adhesins that localise to the attachment organelle bind host molecules. The data presented here suggests that cleavage fragments of Mpn142 function as adhesins that bind a wide range of host molecules.

Mpn142 comprises 1218 amino acids and is cleaved generating 40 (P40) and 90 kDa (P90) fragments on the surface of *M. pneumoniae* [33,52,96,97]. Mutants that cannot synthesize Mpn142 are unable to localise the major adhesin, P1 to the tip of the attachment organelle and lose the ability to adhere to surfaces [40,47]. In addition, P90 forms a 480 kDa protein complex in a 1:2 molar ratio with P1 and forms an appendage that allows *M. pneumoniae* to glide across surfaces [37]. These observations suggest that P90 and P40 function as adhesins either directly via interactions with receptors on cell and abiotic surfaces or via collaborative interactions with P1. We show here that tryptic peptides spanning 15 fragments of Mpn142 were identified by LC-MS/MS in size fractionated lysates of *M. pneumoniae* (Figure 2). Previous studies show that a cleavage event at position 455 in Mpn142 was seminal to the creation of the dominant cleavage fragments P90 and P40. Edman degradation of the N-terminus of P90 generated the sequence ^455^RAGNSSETDAL^465^ [33]. Another cleavage event at position 26 removes the leader sequence in the N-terminus which correlates with the first peptide we find for P40 [34]. As such, P40 was thought to span amino acids 26–454 (theoretical mass of 44.9 kDa) and P90, amino acids 455–1218 (theoretical mass of 82.8 kDa). However, during SDS-PAGE, P40 migrates with a mass of 35–40 kDa [33,52] prompting speculation that further cleavage events occur in the proposed P40 sequence [11]. In our study, the largest fragment spanning the N-terminal region of Mpn142 spans amino acids 26–368. A dimethyl-labelled peptide ^369^ASDTATFSKYLNTAQALHQMGVIVPGLEKWGGNNGTGVVASR^410^ (peptide N12; Table 1) commencing at amino acid 369 indicated that P40 spans amino acids 26–368 (theoretical mass 36.2 kDa), a size consistent with earlier studies of P40 [34]. In support of this hypothesis, we rarely identified tryptic peptides that mapped in the disordered region spanning amino acids 369–444 suggesting this region is readily accessible to different proteases. Notably, we identified a series of six semi-tryptic peptides spanning amino acids 253–308 and eight semi-tryptic peptides spanning amino acids 368–414 that differed by the sequential loss of a C-terminal amino acid (Table 1; peptides S1–S6 and peptides N5–N12, respectively) indicating that *M. pneumoniae* displays carboxypeptidase activity on the cell surface. There is evidence of this clipping of terminal amino acids in *M. hyopneumoniae* [69]. The N-terminal peptide consistently identified in P90 (^446^AVTVVAGPLR^455^) started nine amino acids upstream of the predicted start site defined by the sequence ^455^RAGNSSETDAL^465^ (Table 1: peptide N13). A second semi-tryptic peptide ^447^VTVVAGPLR^455^ was identified (Table 1: peptide N14) in this region suggesting aminopeptidase(s) that target hydrophobic amino acid residues are active on the surface of *M. pneumoniae*. This hypothesis was supported by the identification of a series of six dimethyl-labelled peptides and one semi-tryptic peptide from 1190 to 1218, each one differing by the loss of a single N-terminal amino acid (Table 1; peptides N19–N24 and peptide S10). Collectively, our data suggests that P90 commences at position 446 and that peptidase activity can alter the N-terminus generating size variants of P90. We characterised N-terminal dimethyl-labelled peptides by LC-MS/MS as a method to define precise cleavage events in Mpn142. Using this approach, we identified 17 peptides each indicating the start of a distinct proteoform derived from Mpn142 (Table 1, peptides N2–N5, N12–N24). The location of these peptides is consistent with processing sites in Mpn142 shown in Figure 2 and tryptic peptides that map to all 15 fragments of Mpn142 recovered by affinity chromatography. Other cleavage events were mapped by characterising truncated C-terminal and other semi-tryptic peptides (Table 1).

Notably, 21 peptides spanning amino acids 43–1158 of Mpn142 were identified when freshly cultured whole cells of *M. pneumoniae* were exposed to mild proteolysis with trypsin. While these data provided further evidence that Mpn142 is on the surface of *M. pneumonia*, we noted that 16 of the 21 peptides were not tryptic. Further analysis showed that eight peptides were semi-tryptic at the N-terminus, eight peptides were semi-tryptic at the C-terminus and five peptides were tryptic. This data is consistent with the presence of peptidase activity on the cell surface. Consistent with these data, we identified a range of proteases on the surface of *M. pneumoniae* in surfaceome studies (our unpublished data). Interestingly, the peptide ^381^TAQALHQMGVIVPGLEK^397^ is the only peptide we identified in our study to reside within the disordered region spanning amino acids 214–453. The identification of six dimethyl-labelled peptides and one semi-tryptic peptide (Table 1; peptides N19–N24 and peptide S10) suggests that the C-terminus of Mpn142 spanning amino acids 1190–1218 is released into the extracellular milieu. Further work is needed to determine if the peptide ^1190^AANNAAPKAPVKPAAPTAPRPPVQPPKKA^1218^ and derivatives of it have adhesive or immune-modulatory functions.

## Supplementary Materials

Supplementary File 1
