# Supplementary material for: P40 and P90 from Mpn142 are Targets of Multiple Processing Events on the Surface of Mycoplasma pneumoniae
Source: Proteomes. 2015 Dec 16;3(4):512–37. doi: 10.3390/proteomes3040512 (PMC5217387; doi:10.3390/proteomes3040512)
Supplement: Supplementary File 1 [file proteomes-03-00512-s001.zip › proteomes-03-00512-Supplementary.pdf]

Supplementary Materials

Table S1. Predicted 2D PAGE locations of Mpn142 fragments against the observed 2D PAGE locations.

Fragment #	Predicted Mass (kDa)	Predicted pI	Observed Mass (kDa)	Observed pI
**1**	30.59	8.86	35	>7, <6, 6.9, 7.8, 8.5–8.7
			37–50 *	4.7, 6.0, 6.6, 8.0, 8.5, 9.5, 9.7
**2**	83.57	5.29	80	4.9, <6, 6.2, 6.5
			80 *	4.6–5.1
**3**	36.30	9.28	38–40	6.5, 6.8, 7.0, 7.5
**4**	35.57	4.83	Not identified	Not identified
**5**	48.01	6.94	Not identified	Not identified
**6**	16.60	9.22	Not identified	Not identified
**7**	12.68	6.80	Not identified	Not identified
**8**	21.16	9.73	Not identified	Not identified
**9**	57.05	7.82	60	4.9–5.0
**10**	62.42	4.77	60	4.9–5.0
**11**	16.37	9.87	14	>7
**12**	25.60	9.38	Not identified	Not identified
**13**	36.67	4.91	Not identified	Not identified
**14**	9.24	4.55	Not identified	Not identified
**15**	53.02	7.91	Not identified	Not identified

Predicted mass and pI was calculated by ProtParam. * indicates that the fragment was identified 2D gels of surface biotinylated proteins. # indicates number.

Table S2. Blast analysis of Mpn142 fragments.

Fragment#	Homology (UniProt Accession #: Name)	Organism	Identity (%)
**1**	D5FY38: Adhesin	*Mycoplasma genitalium*	40.4
**2**	D5FY44: Adhesin	*Mycoplasma genitalium*	58.0
**3**	D5FY38: Adhesin	*Mycoplasma genitalium*	39.0
**4**	D5FY44: Adhesin	*Mycoplasma genitalium*	58.0
**5**	E9JMQ6: Adhesin	*Mycoplasma genitalium*	67.6
**6**	D5FY38: Adhesin	*Mycoplasma genitalium*	56.1
**7**	E9JMP4: Adhesin	*Mycoplasma genitalium*	59.5
**8**	E9JMQ6: Adhesin	*Mycoplasma genitalium*	74.8
**9**	E9JMQ6: Adhesin	*Mycoplasma genitalium*	66.5
**10**	D5FY44: Adhesin	*Mycoplasma genitalium*	59.9
**11**	E9JMQ6: Adhesin	*Mycoplasma genitalium*	75.3
**12**	E9JMQ6: Adhesin	*Mycoplasma genitalium*	71.4
**13**	E9JMP1: Adhesin	*Mycoplasma genitalium*	62.5
**14**	E9JMQ0: Adhesin	*Mycoplasma genitalium*	65.4
**15**	E9JMQ6: Adhesin	*Mycoplasma genitalium*	66.0

Proteins listed were the top Blast non-*M. pneumoniae* hits. # indicates number.

© 2015 by the authors; licensee MDPI, Basel, Switzerland. This article is an open access article distributed under the terms and conditions of the Creative Commons Attribution license (http://creativecommons.org/licenses/by/4.0/).
